# Increasing the Execution Speed of Containerized Analysis Workflows Using an Image Snapshotter in Combination With CVMFS

**DOI:** 10.3389/fdata.2021.673163

**Published:** 2021-05-11

**Authors:** Simone Mosciatti, Clemens Lange, Jakob Blomer

**Affiliations:** CERN, Experimental Physics Department, Geneva, Switzerland

**Keywords:** Kubernetes, CVMFS, containers, Docker, workloads, high-energy physics

## Abstract

The past years have shown a revolution in the way scientific workloads are being executed thanks to the wide adoption of software containers. These containers run largely isolated from the host system, ensuring that the development and execution environments are the same everywhere. This enables full reproducibility of the workloads and therefore also the associated scientific analyses performed. However, as the research software used becomes increasingly complex, the software images grow easily to sizes of multiple gigabytes. Downloading the full image onto every single compute node on which the containers are executed becomes unpractical. In this paper, we describe a novel way of distributing software images on the Kubernetes platform, with which the container can start before the entire image contents become available locally (so-called “lazy pulling”). Each file required for the execution is fetched individually and subsequently cached on-demand using the CernVM file system (CVMFS), enabling the execution of very large software images on potentially thousands of Kubernetes nodes with very little overhead. We present several performance benchmarks making use of typical high-energy physics analysis workloads.

## 1. Introduction

A software container is a packaged unit of software that contains all dependencies required to run the software independently of the environment in which the container is executed. Since the virtualization of the environment takes place at the operating-system (OS) level, the overall resource overhead is small, in particular compared to virtual machines that virtualize at the hardware level. Nevertheless, the containers run as isolated processes. Software containers are therefore widely used in industry but also in scientific high-throughput computing.

In the context of reproducible scientific analysis workflows, containers play a very important role. In this study, we in particular focus on high-energy physics (HEP) applications. For example, the physics experiments at the Large Hadron Collider (LHC) (Evans and Bryant, 2008) particle accelerator at CERN have been running for more than a decade. Scientific Linux CERN 5 (Scientific Linux CERN, 2021), the OS used at the beginning of data-taking campaigns in 2009, has reached its end-of-life years ago and thus does not receive security updates anymore. Even the OS used for last data-taking campaign in 2015–2018, Scientific Linux CERN 6, is not maintained since December 2020. Therefore, no large-scale installations running these systems exist anymore, and software not ported to a more recent OS can only be executed at scale using containers. Furthermore, central data set production jobs using the Worldwide LHC computing grid (The Worldwide LHC Computing Grid, 2021) are largely running using software containers by now, for example for the CMS experiment (CMS Collaboration, 2008).

One recurring issue with the execution of containerized workloads is the slow startup time of a container compared to directly running from the file system of the computing node. The container image, which usually contains a complete Linux distribution, the actual application binaries of interest and further package dependencies, first needs to be downloaded to the node's file system. The size of such an image typically ranges from tens of megabytes for a base OS image to several gigabytes for complex applications, therefore also causing a significant network load. There are effectively two ways to mitigate this issue: one can provide a pull-through cache or local registry (Registry as a pull through cache, 2021) to store the container images on machines in the local network close to the execution node, or one can “lazy-pull” (Harter et al., 2016) the images. Lazy-pulling means that image data gets downloaded only as necessary. In this paper, we effectively combine these two approaches, quantifying performance gains for typical HEP workloads. In section 2 the methodology and overall setup are described. Results are presented in section 3, followed by a discussion in section 4.

## 2. Methods

The goal of our study is to evaluate the feasibility of executing arbitrary containerized workloads using a novel lazy container pulling approach that makes use of a caching system that is by now commonly used for software distribution in HEP and related areas. The tools and methodology used for that purpose are described in the following.

### 2.1. Container Workloads Analyzed

We analyze a range of different workloads ranging from a base OS image over programming language images to typical HEP analysis images. All these images are available on Docker Hub (Docker Hub, 2021) and summarized in Table 1. The CentOS 7 Linux distribution is currently the default OS for computing clusters at CERN (Linux @ CERN, 2021). This image is therefore commonly used for simple containerized workloads that only need access to a shell. At the time of writing, most HEP software is written in C++ and/or Python, which is why these images reflect a realistic use case for experiment-independent software needs. This is similarly the case for the ROOT image, which effectively builds on top of the C++ and Python images: ROOT is an open-source data analysis framework (Brun and Rademakers, 1997), which includes a C++ interpreter as well as Python bindings, and constitutes the basis for a vast number of HEP software frameworks. For all four images, the “workload” is to print “hello” onto the console. For the CentOS image, the echo command is used, while in case of the Python image the Python interpreter is used for printing. For the gcc image, a C file is created on the fly, compiled, and the resulting binary executed. For the ROOT image, the integrated C++ interpreter is used. In addition to the base images discussed, an additional image that contains parts of a realistic Higgs boson physics analysis (Jomhari et al., 2017) is used. This image contains a full CMS software (CMSSW) (CMSSW Software, 2021) release, version CMSSW_5_3_32. The C++ analysis code is already compiled, since it would typically be used to run over thousands of input files in parallel. For the benchmarking purposes here, the code analyzes a single file in a CMS-specific format, which has previously been copied to the computing node for local file system access.

**Table 1:** Container images used in the analysis.

**Image name**	**Size [MB]**	**Description**
centos:7.9.2009	76	CentOS 7.9 base image
python:3.9	338	Python 3.9 base image
gcc:10.2.0	427	GNU Compiler Collection 10.2.0 base image
rootproject/root:6.22.06-ubuntu20.04	551	ROOT 6.22.06 based on Ubuntu 20.04
clelange/cms-higgs-4l-full:latest	4,519	CMS Higgs boson analysis example

*The string after the colon in the image name indicates the image version/tag. The size corresponds to the compressed image size in the container registry*.

### 2.2. Kubernetes

The current de-facto standard to leverage and manage a large number of containers is the Kubernetes container-management system (Burns et al., 2016). Kubernetes exposes an API for this purpose, which can also be used to monitor events in the computing cluster. For the purpose of this study, we set up a Kubernetes cluster consisting of one control-plane node and four worker nodes. We then create Jobs using the batch/v1 API to create the container workloads described in section 2.1 on selected worker nodes. The Kubernetes version used is v1.20.2. One of the main reasons we opted for a Kubernetes cluster is that this approach is easily scalable to larger workflows and would also be applicable to public compute clouds. Furthermore, several WLCG sites are in the process of moving to Kubernetes, or have already done so.

### 2.3. The CernVM File System and the WLCG

The caching mechanism used in the following is based on the CernVM file system (CVMFS) (Blomer et al., 2011, 2020). CVMFS is a distributed file system, designed to distribute HEP experiment software onto virtual machines and batch-compute worker nodes. It features a read-only client that allows different machines to access the software installed in CVMFS using an on-demand download mechanism, with which only the files required are downloaded and consequently cached on the machine until the machine's cache size limit is reached. For the distribution of files, a standard HTTP transport is employed, which allows exploitation of a variety of web caches such as a local squid proxy cache (2020b). CVMFS moreover features content-addressable storage for files, which enables the deduplication of files at both storage and cache level, allowing different workloads to benefit from a single shared cache.

CVMFS is widely deployed on the WLCG and is therefore accessible on all major WLCG sites. This implies that any software distributed via CVMFS is also accessible by the whole WLCG, which significantly simplifies software distribution. CVMFS has been one of the cornerstones of software distribution inside the WLCG for several years, and at this point is a hardened and very reliable way to distribute files to the WLCG sites.

### 2.4. The CVMFS Image Snapshotter

While Kubernetes can be used to manage containers, it does not spawn containers itself. For pulling and running container images, it relies on the so-called container runtime. One of those containers runtimes is containerd (2020a). The containerd runtime has a plugin mechanism that allows different plugins to modify containerd's internal working mechanisms. Among those modifiable components, the so-called snapshotter is the one that mounts the layers of a container image and thus makes the container's file system available to the container runtime.

In this work, we make use of a novel containerd plugin, the CVMFS snapshotter (CVMFS containerd snapshotter, 2021), to provide image layers previously ingested into CVMFS to the container runtime. Using CVMFS, the CVMFS snapshotter can mount the layers without downloading any content, only making use of the file metadata provided by CVMFS as opposed to the actual file data.

Once all the layers have been mounted, the container runtime has access to the full file system of the container image. Only when the container runtime starts to access and load files, for instance the bash executable, the CVMFS client fetches the actual files. The CVMFS client will hereby first attempt to load the files from the local cache. If the file is not present, the file is downloaded via the network.

The containerd snapshotter plugin interface that is used by the CVMFS snapshotter has originally been developed in connection with the Stargz Snapshotter (2021). The Stargz snapshotter introduces a backwards-compatible change to the OCI image format (Open Container Initiative, 2021) that facilitates lazy pulling. However, we did not use the Stargz approach here directly, because at the time of writing most container images had not yet been migrated to the new image format. Furthermore, the Stargz snapshotter is not designed to use third-party caches for content distribution. Instead, all clients have to fetch content directly from a centrally managed source. This limits its scalability in federated infrastructures that are typically deployed for scientific collaborations.

### 2.5. DUCC – The Daemon that Unpacks Container Images into CVMFS

The CVMFS containerd plugin relies on a defined structure of a particular CVMFS repository, which is called unpacked.cern.ch. This CVMFS repository is devoted to providing container images to the WLCG. Using the CVMFS service DUCC, “the Daemon that Unpacks Container Images into CVMFS” (Working with DUCC and Docker Images (Experimental), 2021), container images of interest are automatically downloaded and unpacked into a local file system. The container images are then provided in the unpacked.cern.ch repository in two different formats: as a flat file system, which can be used by the Singularity container platform, which is widely used on the WLCG, or as a set of layers, to be used by the containerd snapshotter or other OCI-compliant runtimes.

In the latter case, each layer is unpacked into a specific directory, referred to as the layer digest. This ensures that lower layers of a container image are not overwritten by higher (more recent) layers, and thus enables sharing of layers between images. This is particularly relevant when the same base images are used or changes to container images are only incremental. Moreover, this file system structure allows for an immediate lookup of the layer location given only its digest.

### 2.6. Compute Cluster Setup

The compute cluster used in this work consists of six virtual machines (VMs), each equipped with four-core Intel Xeon Skylake processors with 2.3 GHz clock, 7.1 GB RAM, and local 40 GB SSD storage. The VMs are deployed in the same networking zone within the CERN network to minimize possible effects caused by network latency (the ping response time is usually below 0.5 ms). As described in section 2.2, five of the machines make up the Kubernetes cluster. These machines run Ubuntu Linux 20.04.2 LTS (Ubuntu Linux, 2021) with Kubernetes installed on top with the Calico container network interface v3.17.2.

The sixth VM runs CentOS Linux 8.2 and serves as both the local squid proxy cache server and the local container registry (registry:2.7.2 on Docker Hub). Both services are containerized and deployed using podman v2.0.5 (Podman, 2021). The images described in section 2.1 used for the benchmarks are pushed into the local container registry so that bandwidth limitations only have minor effects. For the same reason, files in CVMFS required for the execution of the benchmarking jobs are pre-cached on the squid server. The bandwidth between the Kubernetes nodes and this VM was measured to be around 6.5 Gbits/s.

Two of the Kubernetes worker nodes are configured to use vanilla containerd v2.4.2 and set to pull the container images from the local container registry. We refer to these machines as “legacy” in the following. The other two worker nodes use the some containerd version, but additionally have the CVMFS unpacked.cern.ch repository mounted with a proxy configuration pointing to the squid proxy cache server. Furthermore, containerd is configured to use the CVMFS snapshotter v0.1.1 and we refer to the machines as “snapshotter” machines.

### 2.7. Benchmarking Procedure

Benchmarking is performed accessing the Kubernetes API via the Kubernetes Golang client library, which allows us to observe the pod (i.e., the container) life-cycle details and transitions in the cluster. For each image and worker node combination, ten measurements are performed. For each run, we evaluate the image pull time, the time for the pod to be ready after pulling, and the overall run time. Furthermore, we extract the amount of data downloaded to the node.

Before starting each measurement, the node's image cache as well as the CVMFS cache for the snapshotter machines is wiped. For the legacy machines, the container image of interest therefore has to be downloaded from the local registry each time. Similarly, for the snapshotter machines, the container manifest needs to be downloaded each time and the node's CVMFS cache be populated accordingly. The measurement is started as soon as the pod is successfully scheduled on the respective node. We record the time again once the pod starts executing the actual workload and stop the time when the pod is succeeded, which means that execution of the workload has completed successfully. Failed runs are discarded. We found that the reason runs failed was that we hit the Docker Hub download rate limit when obtaining the image manifest, which means that in the future other/dedicated container registries should be used instead. The data downloaded is taken as the compressed image size for the legacy machines, and for the snapshotter machines we determine the data downloaded from the squid proxy using the cvmfs_talk command, which is part of the CVMFS client installation. Furthermore, measurements without deleting the image cache are performed to evaluate possible overhead.

## 3. Results

Results are presented separately for container startup time and the measured data transfer.

### 3.1. Container Startup and Run Times

**Figure 1:**
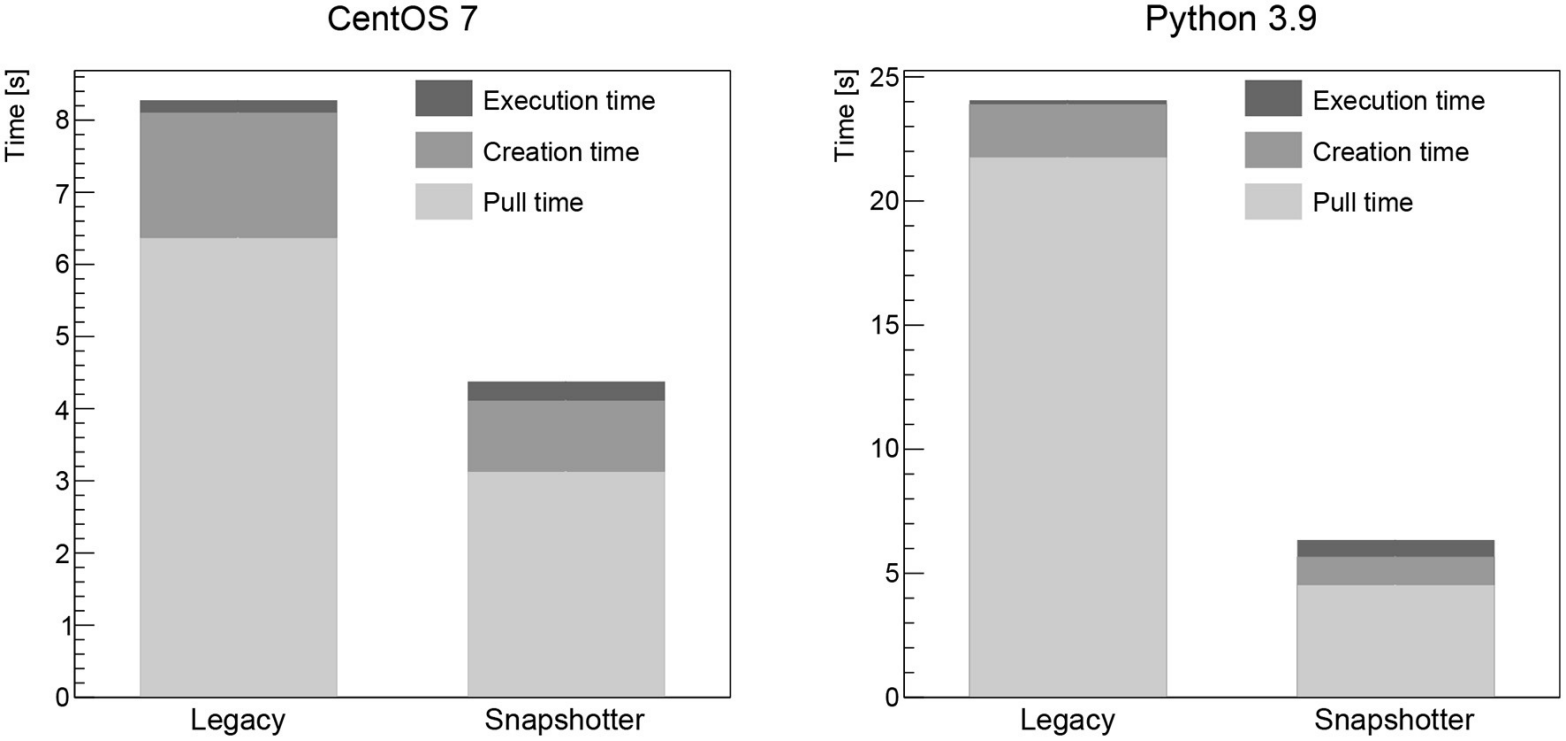
Summary of the overall run time of the CentOS **(Left)** and Python **(Right)** workloads analyzed split into the container image pull time, the time to start the job after the container is ready as well as the time for the actual workload comparing the legacy and the snapshotter approaches.

**Figure 2:**
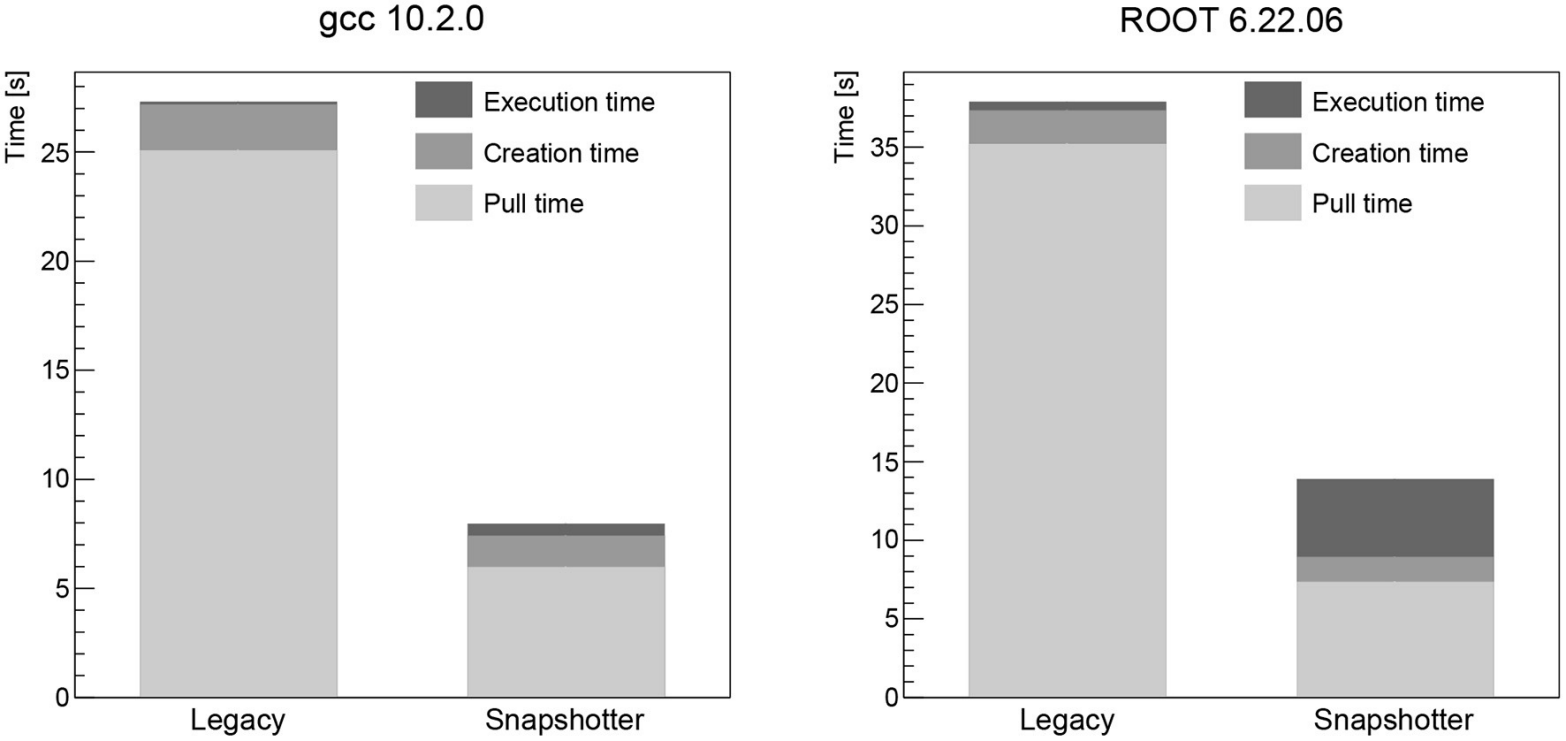
Summary of the overall run time of the gcc **(Left)** and ROOT **(Right)** workloads analyzed split into the container image pull time, the time to start the job after the container is ready as well as the time for the actual workload comparing the legacy and the snapshotter approaches. For the ROOT workload, a large fraction of the image data is required and downloaded during the execution stage, significantly increasing the execution time.

**Figure 3:**
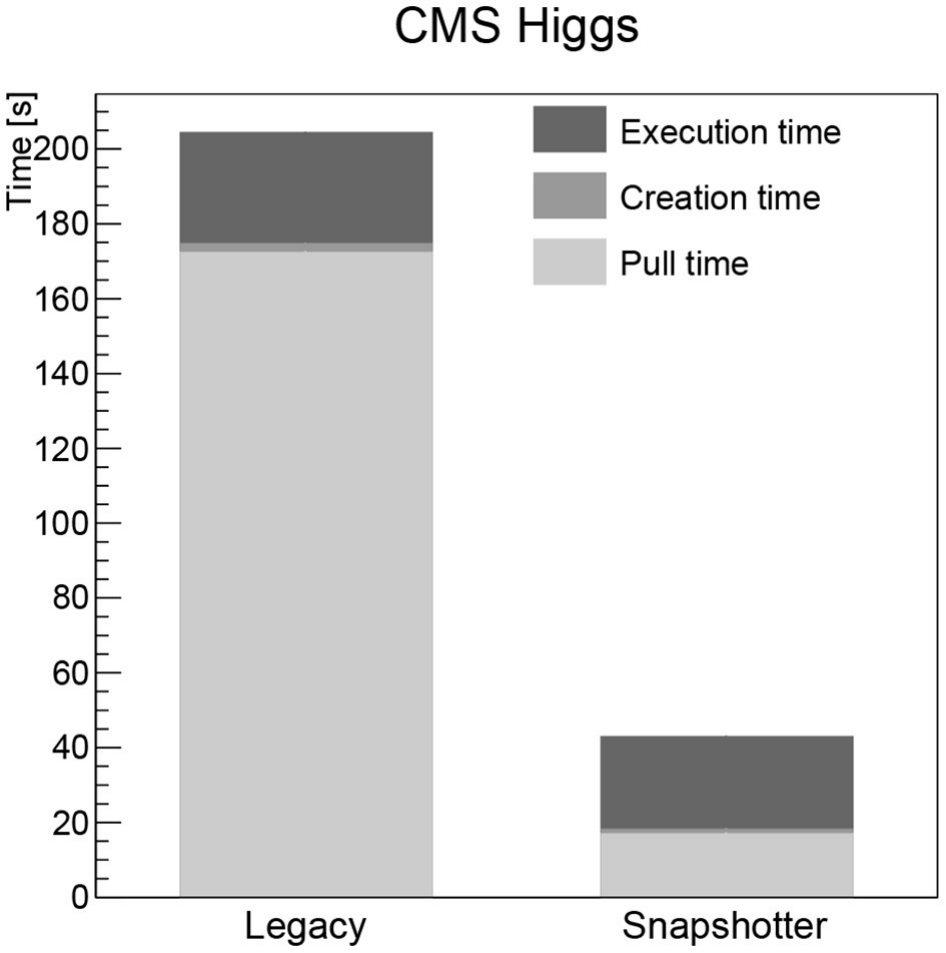
Summary of the overall run time of the CMS Higgs boson analysis workload analyzed split into the container image pull time, the time to start the job after the container is ready as well as the time for the actual workload comparing the legacy and the snapshotter approaches.

The pull time when using the CVMFS snapshotter is found to be a few seconds for all images, increasing with image size. The largest image, the Higgs boson analysis image, takes 18 s to start (see **Figure 3**). When using the legacy machines, the pull time scales linearly with the image size, as expected. The container creation time is negligible for both approaches. The workload execution time is a few seconds or less for the simple workloads (CentOS, Python, gcc, see Figures 1, 2 left) and just below 30 s for the Higgs boson analysis with no significant differences between legacy and snapshotter. However, for the ROOT image (see Figure 2 right), we observe that the workload execution time is about a factor five shorter for the legacy machines than for the snapshotter ones. In this case, a large number of additional files required for the workload that were, however, not needed for the container startup, are downloaded during execution. The container startup and run times are summarized in Figures 1–3. When executing the jobs with a pre-populated cache on the worker nodes, the pull time is found to be around 1 s or less for both approaches.

### 3.2. Data Transferred

**Figure 4:**
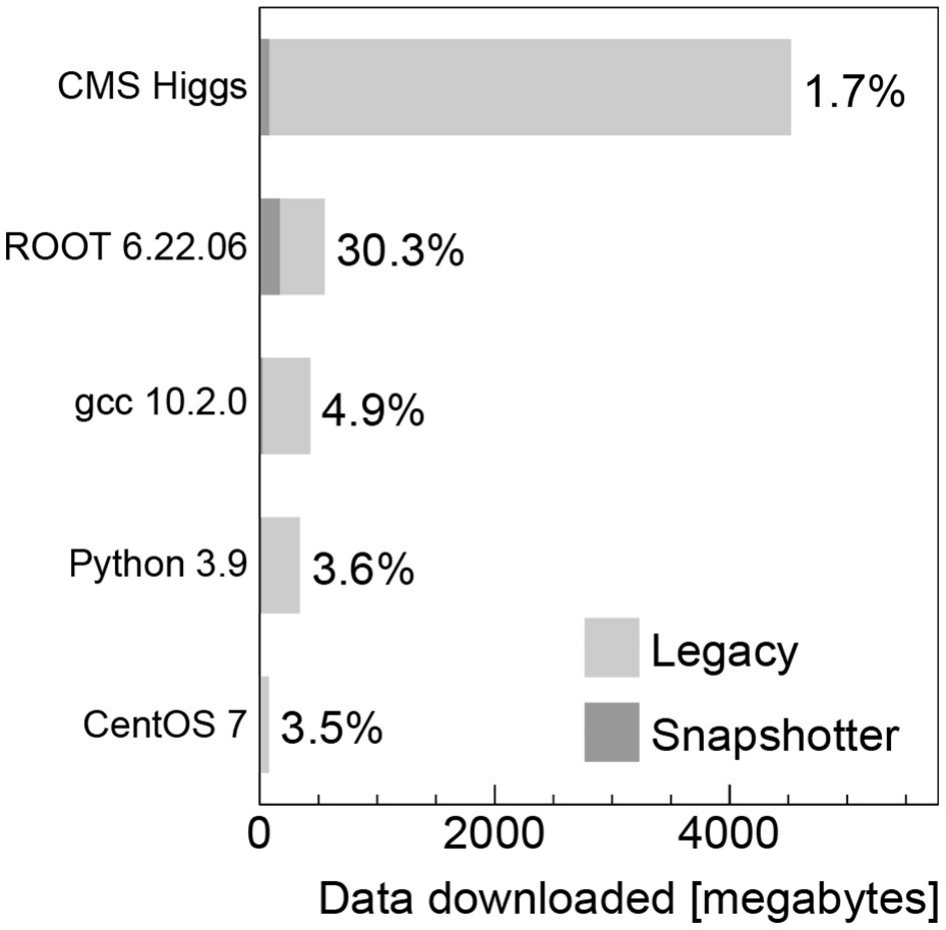
Data downloaded from the container registry and CVMFS for the legacy and snapshotter machines, respectively, in megabytes for the container images analyzed. The percentages indicate the fraction of data downloaded when using the CVMFS snapshotter compared to the full compressed image in the registry.

The amount of data downloaded from the local container registry when using the legacy machines and the data downloaded from the proxy cache server when using the CVMFS snapshotter machines is shown for the container images analyzed in Figure 4. The data downloaded to the legacy machines corresponds to the respective compressed image sizes given in Table 1, since the complete image needs to be downloaded before the job can start. In contrast to that, for the snapshotter machines, only the actual required data to execute the workload has to be downloaded. We observe that the data actually used is typically at level of a few percent, but increases up to 30% for the ROOT image. When running the jobs without deleting the image cache, the amount of data transferred is at the kilobyte level, because only the image manifest is downloaded.

## 4. Discussion

Our studies show that the snapshotter approach using lazy image-pulling in combination with aggressive caching using CVMFS is significantly faster than the legacy approach of completely downloading the container image before execution for all evaluated workloads. The time for the container to start is reduced by at least a factor two for the smallest CentOS image (see Figure 1 left), and this factor increases as a function of the image size. Furthermore, the amount of data downloaded is reduced dramatically, since in most cases only a fraction of the image's data is needed for workload execution. This is particularly important for HEP workloads, which often consist of a large number of jobs that are run in parallel on a large number of nodes. Provisioning a quasi-local cache such as CVMFS is therefore mandatory to avoid bandwidth problems.

For images that use a large fraction of the image data, i.e., in our analysis the ROOT image (see Figure 4), an increase in the workload execution time is found when using the snapshotter (see Figure 2 right), which downloads the required data on the fly during the execution stage as opposed to having the full image available already. While this is somewhat a worst-case scenario for the snapshotter approach, the overall run time is still significantly shorter. We think this can be addressed by pre-fetching bundles of files in the CVMFS client. Such file bundles can be automatically created by tracking the files needed to start a container, and possibly also executing the image's main binary. The implementation and evaluation of a CVMFS pre-fetch capability is subject to future work.

If one optimized the container image by reducing the size overhead and thus increased the fraction of data used, the performance gains of the snapshotter compared to the legacy approach could decrease. However, this would make each image very use-case specific and would also require additional work when creating the image. An alternative approach could be to schedule jobs with the same base layers on the same execution nodes. However, due to the high variety and large number of workloads, this could be challenging to achieve and would need more work on the job scheduler side. Overall, the snapshotter approach presented in this work performs and scales significantly better than previous approaches, would not require any changes to current standard container image build processes, and integrates with modern compute platforms. At the time of writing, the CVMFS snapshotter is actively used for smaller Kubernetes installations at CERN. Furthermore, several WLCG sites are evaluating the use of Kubernetes on-premise as well as on public compute clouds for scalability, for which the CVMFS snapshotter will be an attractive tool.

## Data Availability Statement

The raw data supporting the conclusions of this article will be made available by the authors, without undue reservation.

## Author Contributions

SM is the main developer of the CVMFS image snapshotter and contributed to the technical setup as well as the paper writing. CL set up the machines used for benchmarking, performed the measurements, and contributed to the paper writing. JB is the CernVM project lead and contributed to the overall discussion in this paper. All authors contributed to the article and approved the submitted version.

## Conflict of Interest

The authors declare that the research was conducted in the absence of any commercial or financial relationships that could be construed as a potential conflict of interest.
